# Recent Developments in TSPO PET Imaging as A Biomarker of Neuroinflammation in Neurodegenerative Disorders

**DOI:** 10.3390/ijms20133161

**Published:** 2019-06-28

**Authors:** Eryn L. Werry, Fiona M. Bright, Olivier Piguet, Lars M. Ittner, Glenda M. Halliday, John R. Hodges, Matthew C. Kiernan, Clement T. Loy, Jillian J. Kril, Michael Kassiou

**Affiliations:** 1 School of Chemistry, Faculty of Science, The University of Sydney, Sydney 2006, Australia; 2 School of Medical Sciences, Faculty of Medicine and Health, The University of Sydney, Sydney 2006, Australia; 3 School of Psychology and Brain and Mind Centre, Faculty of Science, The University of Sydney, Sydney 2006, Australia; 4 Dementia Research Centre, Macquarie University, Faculty of Medicine and Health Sciences, Sydney 2109, Australia; 5 Brain and Mind Centre, and the Faculty of Medicine and Health, Central Clinical School, The University of Sydney, Sydney 2006, Australia; 6 Institute of Clinical Neurosciences, Royal Prince Alfred Hospital, Sydney 2050, Australia; 7 Sydney School of Public Health, Faculty of Medicine and Health, The University of Sydney, Sydney 2006, Australia

**Keywords:** translocator protein, neuroinflammation, neurodegeneration, microglia, astrocytes

## Abstract

Neuroinflammation is an inflammatory response in the brain and spinal cord, which can involve the activation of microglia and astrocytes. It is a common feature of many central nervous system disorders, including a range of neurodegenerative disorders. An overlap between activated microglia, pro-inflammatory cytokines and translocator protein (TSPO) ligand binding was shown in early animal studies of neurodegeneration. These findings have been translated in clinical studies, where increases in TSPO positron emission tomography (PET) signal occur in disease-relevant areas across a broad spectrum of neurodegenerative diseases. While this supports the use of TSPO PET as a biomarker to monitor response in clinical trials of novel neurodegenerative therapeutics, the clinical utility of current TSPO PET radioligands has been hampered by the lack of high affinity binding to a prevalent form of polymorphic TSPO (A147T) compared to wild type TSPO. This review details recent developments in exploration of ligand-sensitivity to A147T TSPO that have yielded ligands with improved clinical utility. In addition to developing a non-discriminating TSPO ligand, the final frontier of TSPO biomarker research requires developing an understanding of the cellular and functional interpretation of the TSPO PET signal. Recent insights resulting from single cell analysis of microglial phenotypes are reviewed.

## 1. Neuroinflammation in Neurodegenerative Disorders

### 1.1. Neuroinflammation Overview

Neuroinflammation involves a complex multi-stage physiological response triggered by cell damaging processes in the brain. These can include infection, toxins, autoimmunity, trauma, and responses to processes that change neuronal activity. Neuroinflammation is choreographed by the reactive morphology of resident central nervous system (CNS) innate immune glial cells, predominantly microglia and astrocytes, accompanied by a dynamic biochemical cascade of inflammatory factors that modify the CNS microenvironment. Varying degrees of neuroinflammation exist and are dependent on multiple factors such as the duration of the inflammatory response, its course, and the circumstances underlying the primary insult [1]. The neuroinflammatory response is aimed at mitigating triggering factors by evoking CNS immunity to defend against potential harm and to maintain and restore homeostasis. Despite this, neuroinflammation has the potential to be both beneficial and damaging [2,3]. 

The dynamic neuroinflammatory response occurs via activation of microglial pattern recognition receptors (PRRs, including toll-like receptors (TLRs), pathogen-associated molecular patterns (PAMPs) and danger-associated molecular patterns (DAMPs)) [4,5,6] in addition to glial production of a milieu of inflammatory factors including cytokines, chemokines, secondary messengers, and reactive oxygen species [7,8]. Microglia and astrocytes function as both the target and source of these inflammatory cytokines and chemokines [9,10]. If the neuroinflammatory response is not transient or tightly controlled within the CNS, then an uncontrolled, chronic neuroinflammatory response ensues, constituted by the prolonged overactivation of glial cells. This chronic state is deleterious due to the excessive and dysregulated production of pro-inflammatory factors, resulting in prohibited neuronal repair, synaptic impairment, oxidative damage and mitochondrial dysfunction which can lead to or exacerbate neurodegeneration [11,12,13,14]. This highly damaging, chronic response may also result in involvement of adaptive immunity, with the recruitment and infiltration of peripheral immune cells, via disruption of the blood-brain barrier (BBB), which can further initiate neurodegenerative mechanisms [15,16].

Microglia are at the center of the CNS immune response, given they are the chief and resident immunocompetent cells. They conduct a diversity of functions, surveying their surroundings with cellular processes [17,18,19] and adapting quickly to perturbations such as disease, infection, and aging. The activation and adaptation of microglia promotes an inflammatory response that further engages the immune system, involving changes to their morphology and migration to the site of injury to initiate tissue repair within the CNS [19]. While microglia are central to the neuroimmune response, the cellular microenvironment involved in the inflammatory response within the CNS is not limited to microglia, and also includes astrocytes, oligodendrocytes, and peripherally-derived immune cells, the latter constituted by the adaptive immune response. 

Increased glial activation and thus neuroinflammation are components of various pathologies and disease states, including but not limited to cancer, traumatic brain injury, stroke, psychiatric disorders, and neurodegenerative diseases, which are the focus of this review. 

### 1.2. Neuroinflammation in Neurodegenerative Diseases

Despite different etiologies, neuroinflammation is considered a pathological hallmark across the spectrum of neurodegenerative diseases [20,21,22], including Alzheimer’s disease (AD), Parkinson’s disease (PD), amyotrophic lateral sclerosis (ALS), and frontotemporal dementia (FTD). A primary risk factor for these neurodegenerative diseases is advancing in age [23,24] and there is evidence that the brain undergoes ‘inflamm-aging’, progressively acquiring a pro-inflammatory environment across the lifespan, which may contribute to this age-related risk of sporadic neurodegeneration. Specifically, microglia and endothelial cells continue to change phenotypes with age and experience in humans [25,26]. 

Whether neuroinflammation is a primary or secondary event and whether it has an overall beneficial or detrimental effect is hotly debated [3,27,28]. It has been hypothesized that immune activation is an early cause, as opposed to a late consequence of neurodegeneration [10]. In support of this, inflammation has been reported in the early stages of AD prior to the onset of dementia [29,30], similarly in PD, early inflammation in the brainstem has been shown prior to extending cortically with disease progression [31,32] and recently an early role for microglial activation that precedes clinical symptom onset has been shown in FTD [33]. An alternative hypothesis suggests the initial pathological insult, dependent on the phenotype of disease (e.g., amyloid plaques, α-synuclein, tau etc.) may induce an ongoing cytotoxic response, resulting in secondary chronic neuroinflammation, with altered neuronal function in predilection sites also dependent on the phenotype of the disease [28,34,35].

Despite the unknowns regarding timing and precise mechanisms underlying the neuroinflammatory response in neurodegeneration, there is mounting evidence across the spectrum of neurodegenerative diseases indicating that chronic neuroinflammation plays an instrumental role in the pathogenesis and progression of neurodegeneration. Findings from animal models of disease, particularly models of AD and PD, have been extensively reviewed and show early activation of inflammatory processes preceding neurodegeneration and in some instances concurrent with the production and accumulation of intracellular deposits [36,37,38,39]. Post-mortem human brain tissue from patients with neurodegenerative diseases show upregulation of pro-inflammatory cytokines, TLR subtypes, activated microglia, and astrocytes [10,40,41,42,43]. In addition to increased glial activation, post-mortem studies have shown dysregulated inflammatory factors, including, but not limited to, pro-inflammatory cytokines, chemokines, and complement in the blood, serum, and cerebrospinal fluid of disease affected individuals across the spectrum of neurodegenerative diseases [44,45,46,47,48,49]. Perhaps the most compelling evidence to date however, comes from genome-wide association studies (GWAS) reporting the expression of function and disease-causative mutations or associated polymorphisms in genes that are implicated in neuroinflammation and a significant overlap with neurodegenerative diseases and human leukocyte antigen (HLA)-loci (immune system) [23,50,51,52,53,54,55,56,57]. It is also becoming increasingly more understood that this chronic neuroinflammatory component is not restricted to a compromised CNS microenvironment and neuronal function, yet it also involves complex interactions with immunological mechanisms throughout both the central and peripheral nervous systems [8,11,58].

Collectively, investigation across animal studies, post-mortem studies and GWAS, indicate a shift towards a more pro-inflammatory environment in neurodegeneration and further support the importance of neuroinflammation in the pathophysiology of neurodegeneration and its potential as a useful biomarker, and therefore treatment target, across the spectrum of neurodegenerative diseases. 

### 1.3. Monitoring Neuroinflammation as A Biomarker in Neurodegenerative Diseases 

While it is likely that a panel of tools will be necessary to monitor and assess disease status in neurodegeneration, the in vivo monitoring of neuroinflammation in neurodegenerative diseases may provide a number of uses. As mentioned, neuroinflammation is a common finding across multiple neurodegenerative diseases, decreasing the need for development of separate tools for each disease or for multiple imaging of patients with unclear diagnosis. Being able to monitor the trajectory of neuroinflammation as a disease progresses will help understand the degree to which neuroinflammation is a causative or reactive process. Identification of inflammation as early as possible in the primary stages of the neurodegenerative process could enable early delivery of therapeutics, potentially in the prodromal phase of disease [39]. As agents that modulate neuroinflammation are being assessed in clinical trials in diseases such as ALS [59], monitoring neuroinflammation could assist in these trials by allowing tracking of disease progression and providing an indication of therapeutic response [27,60]. Furthermore, clinical trials could be streamlined to assist therapeutic efficacy, by providing insights into the underlying pathophysiology of neuroinflammatory and regulatory pathways [27,39,61]. 

For this to occur, however, an adequate biomarker of neuroinflammation is needed. One protein that has shown promise as a biomarker for neuroinflammation is the translocator protein (TSPO).

## 2. TSPO as a Biomarker For Neuroinflammation

The translocator protein (TSPO), originally named the peripheral benzodiazepine receptor, is an 18 kDa outer mitochondrial membrane protein. It is implicated in a number of functions relevant to neurodegeneration, such as redox homeostasis [62,63,64] and neurosteroidogenesis [65], however the direct role of TSPO in neurosteroidogenesis is the subject of current debate [66,67,68,69]. Clinical interest in TSPO stems from a wide body of evidence that it is upregulated in neuroinflammation, and hence may be a suitable neuroinflammation biomarker. Increased TSPO expression, indexed with immunohistochemistry and TSPO ligand positron emission tomography (PET) imaging, has been observed in a wide variety of animal models of neuroinflammatory conditions, including AD, stroke, brain injury, experimental autoimmune encephalitis, and epilepsy. This increased PET signal is in contrast with the low background expression of TSPO seen in quiescent CNS tissue, and it overlaps both with areas of brain pathology and with areas of increased immunohistochemical staining of TSPO [70,71,72,73,74,75,76,77,78,79,80,81,82]. Furthermore, the TSPO PET signal decreased in successful preclinical trials of novel therapeutics in HD and AD models, suggesting that it could be used to monitor treatment progress in clinical trials [83,84].

These promising preclinical studies prompted clinical studies using TSPO ligands to detect neuroinflammation. Early clinical studies, however, produced mixed results. While several studies using the first-generation radioligand [^11^C]PK 11195 reported higher TSPO PET brain signal in amyotrophic lateral sclerosis, AD, PD, and in brains of people at risk of Huntington’s disease when compared to controls [31,32,85,86,87,88,89,90], other studies using second-generation ligands reported no difference in TSPO PET signal in AD and multiple sclerosis [45,91,92,93,94].

**Figure 1:**
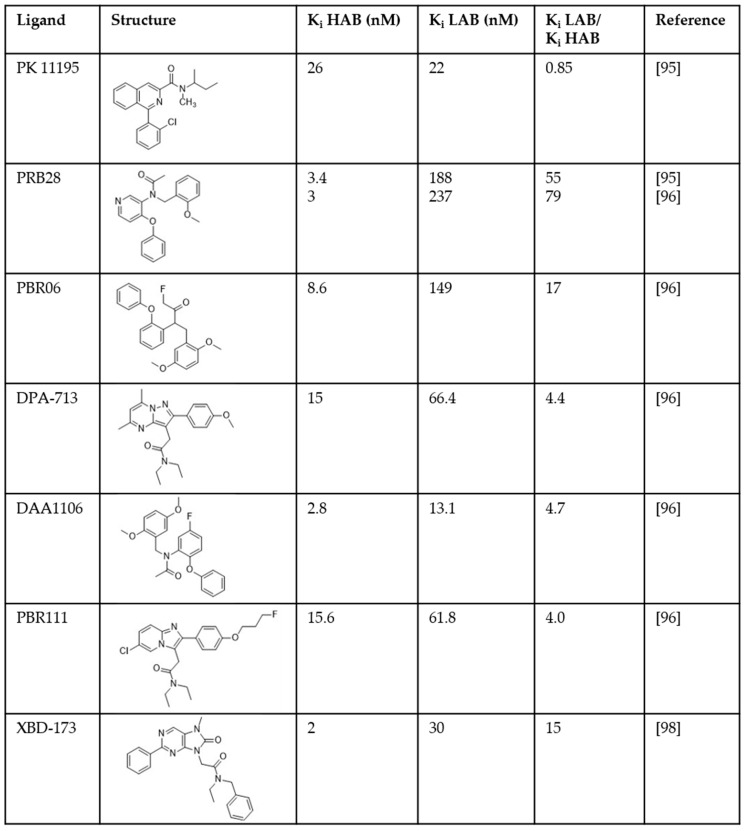
Distribution of affinities of translocator protein (TSPO) ligands measured in homogenates prepared from post-mortem human brain tissue. HAB = high affinity binders; LAB = low affinity binders [95,96,98].

One source of failure in these clinical trials was attributed to large inter-individual variability in PET signal. Second-generation TSPO ligands such as PBR-28, DPA-713, and DAA1106 showed three different binding patterns in tissue from human donors. These ligands bound with high affinity to the tissue from ~50–65% of donors (high affinity binders; HABs) and with low-affinity, or not at all, to tissue from ~5–25% of donors (low affinity binders; LABs) (Figure 1) [95,96,97]. A third group (~30%; mixed affinity binders; MABs) displayed binding of these ligands in a two-site manner for some ligands, or with a K_i_ close to half-way between HABs and LABs for other ligands [95,96,97].

This binding affinity distribution can be predicted by the presence of a single nucleotide polymorphism (SNP) in the *TSPO* gene. This *rs6971* SNP causes a non-conservative substitution of alanine for threonine at the 147th amino acid (A147T) of TSPO. According to the Hapmap database (http://hapmap.ncbi.nlm.nih.gov), it is present in 30% of Caucasians, 25% of Africans, 4% of Japanese, and 2% of Han Chinese. Patients who are HABs to second-generation ligands are homozygous for wild type TSPO, MABs are heterozygous and LABS are homozygous for the A147T TSPO [99,100].

Identification of the impact of A147T TSPO to second-generation ligand binding initiated a change in approach to clinical studies, with studies using second-generation ligands either being stratified for genotype or excluding LABs. This approach showed more consistent success using second-generation radioligands, with higher TSPO PET brain signal found in multiple sclerosis, ALS, mild cognitive impairment, and AD compared to controls [30,101,102,103,104,105,106,107,108,109,110]. These results suggest that TSPO is a good target for development of neuroinflammation imaging agents, however the SNP sensitivity of current TSPO PET radioligands complicate interpretation of results and necessitate genotyping of patients.

## 3. Third-Generation Ligands to Overcome The Challenge of A147T TSPO 

### 3.1. ER176 and GE-180

**Figure 2:**
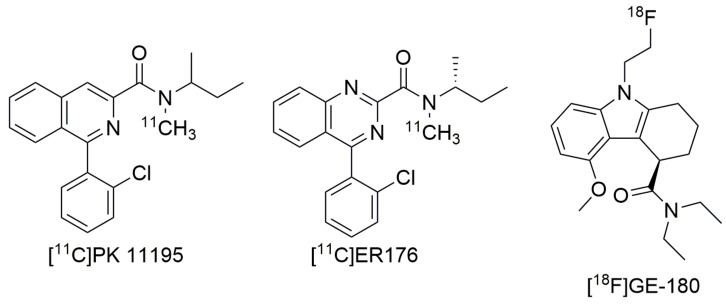
Structures of [^11^C]PK 11195, [^11^C]ER176, and [^18^F]GE-180.

Although stratifying for genotype has opened the door to TSPO PET radioligands being used to monitor therapeutic impact in neurodegeneration clinical trials [111], the ideal situation would see the development of a TSPO ligand with good PET imaging properties, which binds equally highly to TSPO WT and A147T. Two recent ligands, coined ‘third-generation TSPO ligands’, have showed improved detection of TSPO signal in LABs. The first developed out of a strategy to improve the first-generation TSPO ligand PK 11195. In both membrane preparations from human brain tissue and clinical PET scans, [^11^C]PK 11195 does not show binding sensitivity to A147T TSPO (Figure 1; [95]). It is limited as a PET imaging agent, though, by its high non-specific binding, high plasma protein binding and low brain permeability, which make it difficult to accurately quantify [^11^C]-PK 11195 signal in the brain [112]. Zanotti-Fregonara et al [113] examined PK 11195 analogs in a search for one that retained the lack of sensitivity to A147T TSPO but displayed improved imaging characteristics. They identified [^11^C]ER176 (Figure 2), a quinazoline analog of PK 11195, with equally high binding at WT and A147T TSPO in membranes prepared from human brain tissue, and improved lipophilicity (logD decreased from 3.97 to 3.55). In comparison to PK 11195, [^11^C]ER176 shows higher plasma free fractions, improved PET signal in monkey brain, and 80% of this signal can be blocked by administration of unlabeled PK 11195, suggesting specificity [113]. Furthermore, this ligand has the added benefit of increased accuracy of quantification as it does not produce radiometabolites that can enter the brain, unlike other second-generation ligands like DPA-713, PBR28, and PK 11195 [114]. However, despite the lack of sensitivity to A147T in human brain-derived membrane preparations, LABs unexpectedly had an [^11^C]ER176 binding potential (BP_ND_) that was a third lower than HABs during a small first-in-man study [115]. This aside, the BP_ND_ of LABs for [^11^C]ER176, was approximately the same as that for HABs with the widely used [^11^C]PBR28, suggesting that although brain PET signal still needs to be corrected for genotype post-hoc, the BP_ND_ with ER176 should be sufficiently high to detect TSPO across all *rs6971* genotypes, removing the need to exclude LABs [114,115]. As current clinical studies with ER176 have been performed on healthy controls, further evidence of the clinical utility of this ligand will be dependent on its performance in clinical studies using populations displaying neuroinflammation.

PET radioligands based on carbon-11 have a short half-life, restricting their use to facilities that have an on-site cyclotron [116]. Radioligands based on fluorine-18 have a half-life approximately five times longer, increasing their utility [117]. These ligands often have the added benefit of being more metabolically stable, reducing the effect of radiometabolites [118]. A third-generation TSPO ligand that incorporates fluorine-18 is the tricyclic indole [^18^F]GE-180 (flutriciclamide). Preclinical models of middle cerebral artery occlusion and AD showed high [^18^F]GE-180 signal in areas of neuroinflammation, low non-specific binding in unaffected brain tissue, and low contribution to the brain signal from radiometabolites [118,119,120]. In pre-clinical studies, this ligand showed better imaging characteristics than [^11^C]PK 11195 and [^18^F]DPA-714 [119,121], although it did not reflect the extent of microglial activation as accurately as [^11^C]DPA-713 [122]. A small clinical PET study measuring [^18^F]GE-180 signals in relapsing-remitting multiple sclerosis patients identified high signal to-noise ratio in focal lesions, with no significant difference in signal intensity across HABs, MABs and LABs [123]. This lack of sensitivity to the *rs6971* genotype was surprising, as GE-180 drops off in affinity approximately 5-fold at A147T compared to WT TSPO in membrane radioligand binding studies [124]. Follow up studies on multiple sclerosis patients indicated that [^18^F]GE-180 had a low volume of distribution, suggesting low brain penetration [125,126], and it has been suggested that this low uptake generates an image quality that is not high enough to detect differences in binding to LABs and HABs [126]. Alternatively, the high variability in neuroinflammation among the multiple sclerosis patients may have reduced the power to detect significant differences in signal between genotypes [127]. These hypotheses can be examined by correlating [^18^F]GE-180 brain PET signal with immunohistochemical TSPO distribution in brain sections [126,127]. It will also be important to evaluate the potential of [^18^F]GE-180 as a neuroinflammation imaging agent in studies with larger sample sizes to increase their power to detect genotype differences. Another important future study is to compare the quantitative potential of second-generation ligands alongside [^18^F]GE-180 in clinical trials with patients experiencing neurodegenerative diseases that do not show as pronounced BBB breakdown as in multiple sclerosis, as it has been suggested that the high lesion-specific signal in these patients may be due to BBB breakdown allowing entrance of the radioligand into brain parenchyma in damaged areas, but not in intact areas [126]. 

### 3.2. Future Directions

While these novel third-generation ligands may be an improvement on second-generation ligands, as discussed above, they still show sensitivity to A147T TSPO. To advance on these, it will be vital to gain a greater understanding of how the binding requirements of WT and A147T TSPO differ. One approach to examining what drives the loss of affinity of TSPO ligands to A147T TSPO is examining the structural differences between WT and A147T TSPO. A crystal structure of the bacterial Rhodobacter sphaeroides TSPO (*Rs*TSPO), which has 34% homology to human TSPO, shows marked differences between wild type TSPO and the A147T homolog, A139T. TSPO is comprised of five-transmembrane alpha-helices, and the gap between the second and fifth transmembrane segments is smaller in the mutant compared to the wild type *Rs*TSPO [128]. In contrast, a 3D nuclear magnetic resonance characterization of mouse TSPO in complex with PK 11195 found the structures of A147T and WT TSPO closely resembled each other [129,130]. Given this contrast, future work resolving the human TSPO crystal structure in complex with discriminating second-generation ligands will provide valuable information about how the binding requirements of WT and A147T TSPO differ.

Another approach to generating insight about TSPO binding discrimination is high throughput screening of ligand binding at WT and A147T TSPO. This has been performed using a lower-throughput approach with membranes derived from tissue donated by HABs and LABs. This approach has shown endogenous TSPO ligands such as the protoporphyrin X and diazepam binding inhibitor do not discriminate between TSPO WT and SNP, in contrast to synthetic ligands [131], possibly providing a clue about binding requirements. A higher throughput approach using cell lines transfected with WT and A147T TSPO that show similar binding affinities to that generated with donated human tissue, has resulted in a derivative of GE-180 with minimal reduction in binding to A147T TSPO. This ligand, GE-387, crosses into the rat brain in preclinical PET studies, although future studies are required to examine its clinical utility [124]. Structure-activity relationships describing binding affinity differences at A147T vs. WT TSPO generated using this approach are slowly appearing in the literature [124,132]. This approach may yield new ligands that do not discriminate between WT and A147T. Such ligands may also emerge by targeting drug discovery to binding sites other than the PK 11195 binding site. These sites may include the cholesterol recognition amino acid consensus motif (for example, see [133]), or to sites at the interface with TSPO’s association partners, such as the voltage-dependent anion channel and the adenine nucleotide transporter [134]. 

Furthermore, it should be considered that optimizing for affinity determined using TSPO membrane preparations alone may not translate to equal binding potential in PET signals from HABs and LABs. As discussed, despite ER176’s almost identical affinity for TSPO WT and A147T in membrane preparations, LABs still showed lower BP_ND_ than HABs in vivo [115]. One reason for this may be that allosteric TSPO modulators such as cholesterol have a greater effect on second-generation ligand dissociation rate in LABs than HABs or MABs, and if the influence of kinetics and physiologically-relevant modulators are ignored in initial radioligand binding screens on membrane preparations then a pure affinity-based screen may miss this [135]. Secondly, TSPO may be in a different activity state in vivo because of associations with other proteins that are disrupted in the process of membrane preparation for binding assays [114]. Lastly, the production of brain-permeant radiometabolites most effects accurate quantification of PET signal from LABs, and these metabolites may not be generated in radioligand binding studies on membrane preparations [114].

This suggests that a wider drug discovery approach may accelerate development of translatable TSPO PET radioligands that are insensitive to the *rs6971* genotype. In combination with the high throughput affinity screening approach to identify novel leads described above, it may be fruitful to include membrane binding studies measuring kinetics of candidate ligands and conducting binding studies with more physiological buffers containing allosteric modulators like cholesterol. Ex vivo autoradiography assessment of lead ligands should be conducted so affinity of these ligands can be determined for TSPO when it is in complex with other proteins. Finally, the brain-permeability of radiometabolite production should be investigated for identified lead molecules to ensure radiometabolites do not complicate analysis of brain signals. 

## 4. Cellular and Functional Interpretation of TSPO PET Signals

In addition to developing a non-discriminating TSPO ligand, the final frontier of research into neuroinflammation-related TSPO PET imaging requires development of an understanding of the cellular and functional interpretation of the TSPO PET signal. At present, it is not clear whether the presence of TSPO PET signal indicates a predominantly pro-inflammatory or anti-inflammatory state, nor whether the signal indicates the presence of destructive or reparative cell states.

### 4.1. Microglial Phenotypes

A major brain cell type that displays TSPO upregulation in neuroinflammation is microglia [122,136,137,138,139,140,141]. Given microglia adapt to a variety of challenges across their lifespan, it is not surprising that they are a heterogeneous population consisting of multiple phenotypes [142]. Some of these microglial phenotypes enact useful functions, such as maintaining homeostasis, regulating synaptogenesis, and refining synapses [143]. On the other hand, some have a harmful pro-inflammatory function, and some phenotypes perhaps even carry out both useful and harmful actions. Due to this, an understanding of what functional state the TSPO signal in the brain represents will involve discovering which microglial phenotypes upregulate TSPO in neuroinflammation.

**Figure 3:**
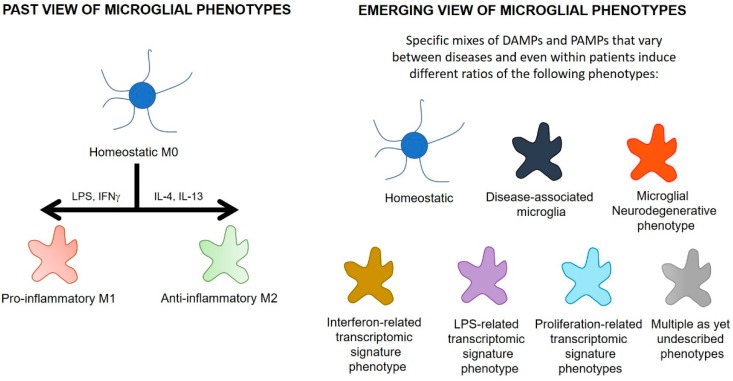
The changing paradigm of microglial phenotypes. DAMPs = danger-associated molecular patterns; PAMPs = pathogen-associated molecular patterns; LPS = lipopolysaccharide; IFNγ = interferon-γ, IL-4 = interleukin-4, IL-13 = interleukin-13.

Until recently, microglial phenotypes were described using similar nomenclature to that which originally defined subpopulations of peripheral macrophages (Figure 3), with classification based on the presence of a limited number of cell markers and cytokines. M0 microglia were described as resting, ramified microglia that maintained homeostasis. Two types of activated microglia were suggested to arise from M0 microglia. A destructive pro-inflammatory phenotype (M1) was suggested to arise on exposure to pathogens, threats or pro-inflammatory stimuli. These M1 microglia were characterized by release of pro-inflammatory cytokines such as IL-1β and TNF-α. A protective anti-inflammatory phenotype (M2) was proposed to emerge on exposure to anti-inflammatory cytokines such as interleukin-4 (IL-4), IL-10, and IL-13 [144].

The phenotypic expression of TSPO has predominantly been examined within this M0/M1/M2 schema. In murine cells, there appears to be a selective upregulation of TSPO in pro-inflammatory M1 microglia. Cultured primary mouse microglia exposed to the pro-inflammatory lipopolysaccharide (LPS), or IL-1β with interferon-γ (IFNγ), upregulated expression of TSPO transcripts, while treatment with the anti-inflammatory IL-4 did not change TSPO transcript levels [145]. Injection of IL-4 into the lateral ventricle of mice did not alter parenchymal TSPO levels, while knockout of peroxisomal multifunctional protein-2 to induce neuroinflammation led to an increase in TSPO expression [145]. Similarly, TSPO expression increased approximately ten-fold in rodent-derived macrophages after a variety of pro-inflammatory stimuli, including LPS and IFNγ [146]. 

In contrast to these consistent findings in mice and rats, there is little consensus about changes in TSPO expression in human microglia and related macrophages, which is in accordance with observations that microglia from human samples often behave differently to rodent microglia [147]. Intraperitoneal delivery of LPS into human volunteers led to an upregulation of TSPO PET signal in the brain, although, as discussed below, this may not solely reflect microglial expression [148]. On the other hand, human microglia grown from a surgical resection did not show a change in TSPO expression even after intense pro-inflammatory induction involving a five-day exposure to granulocyte-macrophage colony-stimulating factor, followed by 1 h IFNγ and 48 h LPS [146]. TSPO expression even decreased after pro-inflammatory stimulation of human macrophages and did not change after anti-inflammatory stimulation [146,149]. Human microglia are known to change physiological properties on removal from the body and subsequent culturing [150,151], so different preparation methods may partly underlie these conflicting findings. 

This conflict may also be a result of the over-simplified theoretical basis of the M0/M1/M2 continuum. This continuum describes microglial phenotypes according to the expression of a few surface markers or cytokines, in response to defined stimuli such as LPS or IL-4. During neurodegenerative diseases, however, microglia are exposed to a complex range of stimuli such as protein oligomers, excitotoxic neurotransmitter levels, reactive oxygen species, and a mix of pro- and anti-inflammatory cytokines. Recent studies using genomic and proteomic techniques have indicated that the cocktail of these stimuli generate a more complex and heterogenous phenotypic distribution of microglia than that previously described in the M0/M1/M2 continuum. These more recent studies have identified over eight different microglial phenotypes (Figure 3) and these are likely to be a small portion of the total number of microglial phenotypes in neurodegenerative diseases [142,152,153,154]. Each phenotype is characterized on the basis of a distinct signature of dysregulated genes. Some of these phenotypes include disease-associated microglia, microglia neurodegenerative phenotype, LPS-related transcriptomic signature microglia, interferon-related transcriptomic signature microglia, and proliferation-related transcriptomic signature microglia (see Table 1 for more detail). Of these newly described phenotypes, disease-associated microglia, microglia neurodegenerative phenotype, and LPS-signature microglia feature an upregulation of TSPO [132,142,144] (Table 1). These studies also suggest some phenotypes of microglia enriched in neurodegenerative conditions do not display TSPO upregulation [142], so the TSPO PET signal might not represent all microglial phenotypes altered in neurodegenerative diseases. As uncharacterized microglial phenotypes start to be described and as research continues to examine the change in TSPO expression on different phenotypes with disease progression, an understanding of the function of the microglia that upregulate TSPO will grow. This holds promise in understanding the functional implications of increased TSPO PET signals.

### 4.2. Astrocytes

As described in Section 1, while microglia are key contributors to neuroinflammation, astrocytes also play a role in neuroinflammation. Given this, immunohistochemistry has been used to examine whether the overexpression of TSPO in neuroinflammation is only seen in microglia, or also manifests on astrocytes. Most studies that have examined this question have found no overlap with TSPO and markers for astrocytes such as glial-fibrillary acidic protein (GFAP) [137,155,156,157], although a small number of studies find GFAP co-localises with TSPO [140,141,158,159,160]. Two of these studies were longitudinal and identified that two weeks after induction of demyelination in a cuprizone-induced demyelination model, TSPO-positive cells were predominantly microglia, whereas six-weeks after demyelination induction TSPO-positive cells were predominantly astrocytes [160]. Thus, it is likely that astrocytes may contribute to the TSPO PET signal in demyelinating pathologies. Future studies that examine the longitudinal overlap between astrocytic markers and TSPO expression in neurodegeneration models will inform the extent to which astrocytes contribute to the TSPO PET signal in other neurodegenerative diseases.

### 4.3. Neurons

Upregulation of TSPO has been reported in the periphery on dorsal root ganglia after sciatic nerve injury [161], and on neural precursor cells and immature neurons in vitro [162]. The majority of reports, however, suggest TSPO is not upregulated on CNS neurons in neuroinflammation [70,162,163,164,165], although one study found an overlap between TSPO immunohistochemical signal and staining for NeuN, a neuronal marker, with a polyclonal TSPO antibody and not a monoclonal TSPO antibody [70]. This highlights the need for careful choice of specific antibodies for investigating the cellular expression of TSPO.

## 5. Conclusions

Neuroinflammation is a common finding in many neurodegenerative disorders. Tracking neuroinflammation through PET imaging may provide an avenue for early delivery of therapeutics, and tracking disease progression and response to novel therapeutics in clinical trials. PET radioligands that target the TSPO have showed promise in the detection of neuroinflammation, however, their clinical utility has been hampered by the presence of a single nucleotide polymorphism producing A147T TSPO at which every disclosed second-generation TSPO PET ligand loses affinity. The recent development of two third-generation TSPO ligands has yielded less-discriminating imaging options, but development of truly non-discriminating ligands will require increased knowledge of differences in A147T and WT TSPO structure, and the development of structure-affinity relationships through high-throughput screening. Furthermore, functional interpretation of the TSPO PET signal may be possible in the future with increasing knowledge of TSPO expression on complex microglial phenotypes and of the temporal differences in the role of astrocytes and microglia in neuroinflammation. 

## Figures and Tables

**Table 1:** Features of new microglial phenotypes from ‘omics studies of neurodegenerative diseases.

Microglial Phenotype	Disease Model	Species	Features	Reference
Disease-associated microglia	Alzheimer’s disease	Mouse, human	Downregulated homeostatic genes (inc *P2YR12*, *P2YR13*, *Tmem119*, *CX3CR1*) and upregulated lysosomal and lipid metabolism-related genes (inc *Apoe*, *Ctsd*, *Lpl*, *Tyrobp*, *TREM2*). *TSPO* is upregulated 2.5x in disease-associated microglia.	[142,152]
Pro-inflammatory disease-associated microglia	Alzheimer’s disease	Mouse	Emerge earlier in the disease. Characterised by pro-inflammatory genes (inc *TLR2*, *Ptgs2*, *Il12b*, *Il1b*), as well as *CD44*, *Kv1.3*, *NFkb*, *Stat1*, *RelA*	[153]
Anti-inflammatory disease-associated microglia	Alzheimer’s disease	Mouse	Upregulation of phagocytic genes (inc *Igf1*, *Apoe*, *Myo1e*), as well as *CXCR4* and *Atf1*.	[153]
Microglial neurodegenerative phenotype	Alzheimer’s disease	Mouse, human	Loss of 68 homoeostatic genes (inc *P2YR12*, *Tmem119*, *CX3CR1*, *CSF1R*, *TGFBR1*) and induction of 28 inflammatory genes (inc *CCL2*, *CSF1*, *Apoe*). *TSPO* is upregulated on these microglia.	[154]
Interferon-related transcriptomic signature microglia	Study analysed a database containing 69 different conditions encompassing neurodegenerative, neoplastic, inflammatory and infectious diseases	Mouse	Dysregulation of many interferon-stimulated genes inc *Irf7* and *Stat2*. Enriched in viral conditions, on LPS-stimulation and in glioma. Also moderately enriched in a number of neurodegenerative disease models.	[152]
LPS-related transcriptomic signature microglia	Study analysed a database containing 69 different conditions encompassing neurodegenerative, neoplastic, inflammatory and infectious diseases	Mouse	Upregulation of inflammation-related genes, including *Ikbke*, *cd44*, *ccl5* and *Tspo*. Enriched on LPS stimulation and in glioma, and a subset of genes are upregulated in neurodegenerative models (*TSPO* did not show much change within the LPS signature in the neurodegenerative models).	[152]
Neurodegeneration-related transcriptomic signature microglia	Study analysed a database containing 69 different conditions encompassing neurodegenerative, neoplastic, inflammatory and infectious diseases	Mouse	Upregulation of genes that regulate how microglia interact with the environment (inc *Bhlhe40*, *Rxrg*, *Hif1a* and *Mitf*), and genes that regulate lysosomal function (inc *Ctsb*, *Ctsl* and *Ctsz*). Induced in most neurodegeneration models.	[152]
Proliferation-related transcriptomic signature microglia	Study analysed a database containing 69 different conditions encompassing neurodegenerative, neoplastic, inflammatory and infectious diseases	Mouse	Dysregulation of 82 genes associated with proliferation (inc *Mki67*, *Cdk1*, *Plk1*). Enriched in viral or neoplastic-related diseases.	[152]

## Abbreviations

A147T TSPO	Ala147Thr translocator protein
AD	Alzheimer’s disease
ALS	amyotrophic lateral sclerosis
BBB	blood-brain barrier
BP_ND_	binding potential
CNS	central nervous system
DAMPs	danger-associated molecular patterns
FTD	frontotemporal dementia
GFAP	glial fibrillary acidic protein
GWAS	genome-wide association studies
HAB	high affinity binder
HLA	human leukocyte antigen
IFNγ	interferon-γ
IL	interleukin
K_i_	affinity
LAB	low affinity binder
LPS	lipopolysaccharide
MAB	mixed affinity binder
PAMPs	pathogen-associated molecular patterns
PET	positron emission tomography
MAB	mixed affinity binder
PAMPs	pathogen-associated molecular patterns
PD	Parkinson’s disease
PET	positron emission tomography
PRRs	pattern recognition receptors
*Rs*TSPO	Rhodobacter sphaeroides translocator protein
SNP	single nucleotide polymorphism
TLRs	toll-like receptors
TSPO	translocator protein
WT TSPO	wild type translocator protein
